# Severe methotrexate toxicity after treatment for ectopic pregnancy: A case report

**DOI:** 10.4274/tjod.80457

**Published:** 2016-12-15

**Authors:** Sunullah Soysal, Gökçe Anık İlhan, Mehmet Vural, Begüm Yıldızhan

**Affiliations:** 1 Marmara University Faculty of Medicine, Department of Obstetrics and Gynecology, İstanbul, Turkey

**Keywords:** Methotrexate, Pregnancy, ectopic, Toxicity

## Abstract

Severe methotrexate toxicity due to medical treatment of an ectopic pregnancy is presented. The feasibility of low-dose use and success of methotrexate makes it the first drug in the medical treatment of ectopic pregnancies. Besides its advantages, it should be used with caution and severe toxicity should be kept in mind.

## INTRODUCTION

Ectopic pregnancy is a serious condition in gynecologic practice. Treatment options for ectopic pregnancy include surgical treatment, expectant management, and medical treatment. Methotrexate (MTX) treatment is preferred for its fewer adverse effects and cost effectiveness^(1)^.

MTX is a folic acid antagonist and is generally used for the treatment of malignancies, autoimmune diseases, and ectopic pregnancies^(2)^. The inhibitory effect on DNA synthesis of MTX is the rationale in the treatment of ectopic pregnancy in which the target is trophoblasts and fetal cells. Ninety percent of MTX given intravenously undergoes renal excretion without any change within 24 hours^(3)^. Low-dose use of MTX gives freedom to a physician to use it frequently in the treatment of ectopic pregnancies. MTX treatment has two protocols; single dose and multiple dose regimens. The single injection of the drug, less follow-up time, lower cost, and no requirement for folinic acid use makes the single-dose protocol the most preferred. MTX is given 50 mg/m^2^ intramuscularly. Levels of beta-human chorionic gonadotropin (β-hCG) are measured on the 4^th^ and 7^th^ day of treatment. A second dose is not needed if the decrease in β-hCG levels is more than 15% between days 4 and 7. A second dose of MTX is needed in 15-20% of cases and only 1% of women who receive the single-dose protocol require a third dose^(4,5,6)^. Adverse effects are generally mild and self-limited; the most common are stomatitis and conjunctivitis. Rare adverse effects include gastritis, enteritis, dermatitis, pneumonitis, alopecia, elevated liver enzymes, and bone marrow suppression. Approximately 30 percent of patients on the single-dose protocol have adverse effects; this rate is lower for patients on multi-dose regimens (40%)^(7)^.

Renal and hepatic diseases, immunodeficiency, active pulmonary disease, and peptic ulcer disease are contraindications for MTX treatment^(8,9,10)^. Herein, an otherwise healthy woman who had a severe adverse event with low-dose MTX treatment is presented.

## CASE REPORT

**Figure 1:**
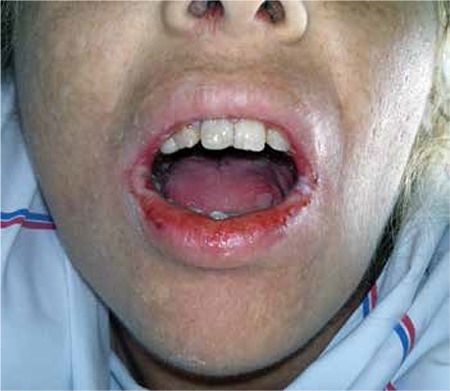
Macular rash on the chest and neck area

**Figure 2:**
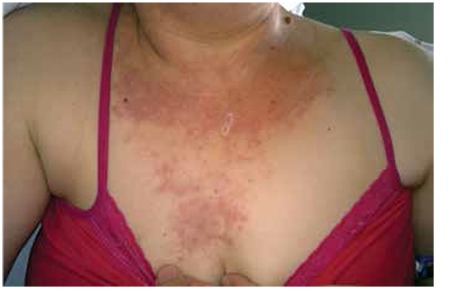
Oral lesions and mucositis detected

A gravida 3 woman aged 38 years was admitted to our hospital with symptoms of pelvic pain, diarrhea, and oral lesions. She had MTX (50 mg/m^2^) treatment when her β-hCG level was 2279 mIU/mL. Seven days after the first dose, a second dose of MTX treatment was given due to elevated β-hCG (2304 mIU/mL) and an ultrasound finding of a 2-cm gestational sac in the right fallopian tube. On day 4 of the second dose, emergency laparoscopic salpingectomy was performed for tubal rupture with hemorrhage. She was discharged from hospital on the first postoperative day and readmitted on the second day with bloody diarrhea (ten times a day), oral lesions, and macular rush on the scalp, neck, and chest regions. No remarkable details were noted in her medical history except MTX treatment. In the physical examination, she was pale and her vital signs and body temperature were in the normal range, and a macular rash on the chest, neck and scalp area (Figure 1) and mucositis in oral mucosa were detected (Figure 2). A blood count revealed hemoglobin (Hb) level of 6.4 gr/dL, the white blood cells number was 2000 /µL, creatinine was 1.76 mg/dL, β-hcg level was 91 mIu/mL. During the clinical follow-up, the general condition of the patient deteriorated. On the same day, her Hb level was 6.2 gr/dL and the white blood cell number was 1600/µL (Table 1). Lesions in the oral mucosa and skin increased and the diarrhea worsened. She was transferred to the intensive care unit. The serum level of MTX was high (more than 2 picograms/L). Calcium folinate infusion, and erythrocyte (4 units), thrombocyte (6 units) replacement and intravenous hydration and nutrition, and broad spectrum antibiotics were started. Sodium bicarbonate was administered via intravenous infusion to increase the excretion of the MTX alkalization of urine. Despite the supplementary treatment and folinic acid treatment, high levels of MTX continued and her cytopenic clinical state worsened. Plasmapheresis was considered for lowering MTX levels because glucarpidase (carboxypeptidase G2) is not available in Turkey. The level of MTX decreased after performing plasmapheresis. The patient’s symptoms and clinical findings regressed. After 18 days of hospitalization (one week intensive care unit) she was discharged from hospital with no symptoms.

## DISCUSSION

MTX is a cheap and minimally toxic drug in low doses and is widely used in patients with non-ruptured ectopic pregnancy in suitable clinical conditions. Before treatment with MTX, a viable intrauterine pregnancy must be excluded; β-hCG levels, renal and liver function tests, and a complete blood count should be checked. MTX treatment has single-dose and multiple-dose protocols. Adverse effects can be seen more frequently in multi-dose protocols^(7)^. The adverse effects of MTX are caused by irreversible inhibition of the enzyme dihydrofolate reductase in purine synthesis. Decreases in blood cells and hemorrhage from the gastrointestinal tract are due to the effect on rapidly dividing cells of the bone marrow and intestinal tract^(11)^. Severe adverse effects are infrequent in MTX treatment for ectopic pregnancy. The potential severe adverse effects of MTX treatment are hepatotoxicity, pulmonary toxicity, risk of infection, myelosupression, and nephrotoxicity. Hepatotoxicity results from direct damage to hepatocytes or in patients with concomitant viral hepatitis. Minor aminotransferase elevations are common but hepatic steatosis, fibrosis, and cirrhosis are seen rarely. For this reason, screening for hepatitis B and hepatitis C virus infection and hepatic enzymes should be performed before initial therapy. MTX is an immunomodulatory but not significantly immunosuppressive agent. Myelosuppression is the major dose-limiting adverse effect of high- dose MTX, but it is infrequent in low-dose therapy. Hematologic toxicity associated with macrocytic red blood cells may be seen, but a more serious abnormality is the development of pancytopenia^(12)^. Therefore, guidelines from the American College of Rheumatology recommend that a routine peripheral complete blood should be performed every four weeks in rheumatoid arthritis treatment^(13)^. Nephrotoxicity due to MTX rarely occurs in treatment with high doses. A slight decrease in creatinine clearance can be seen even at low, weekly doses used in rheumatoid diseases^(14)^. Development of myelosuppression and mucositis such as MTX- related toxicity risk, is highest in patients with prolonged exposure to high levels of plasma MTX concentrations. Glucarpidase, a bacterial enzyme, is used for a rapid decrease of plasma MTX levels, which hydrolyses MTX to its inactive metabolites. The greatest benefit is achieved with glucarpidase when plasma MTX concentrations are high^(15)^. Low-dose MTX is used in the medical treatment of ectopic pregnancy. Severe toxicity is an unexpected condition. In the present case, two doses of MTX were given one week apart but severe toxicity occurred. Compared with other treatment indications of MTX, the dose was very low but the subsequent adverse effects were detrimental and life threatening. Although the patient had no renal insufficiency, a high serum level of MTX was detected. Intensive care unit treatment and folinic acid replacement failed. Fortunately, the patient’s signs and symptoms regressed with plasmapheresis. In review of the literature, severe toxicity due to MTX treatment for ectopic pregnancy was reported in a patient with renal insufficiency but severe toxicity in a healthy woman has not been reported^(2)^. In conclusion, unexpected toxicity with MTX should be kept in mind during use of this simple treatment.

## Figures and Tables

**Table 1:**
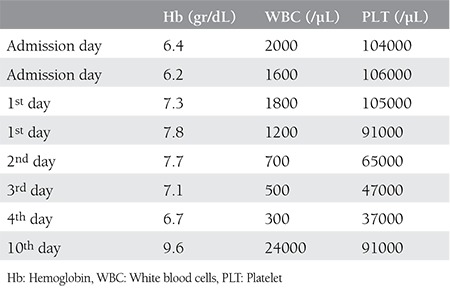
Complete blood count values
